# Evaluating bone strength with finite element analysis for Adolescent Idiopathic Scoliosis (AIS): a case-control study with HR-pQCT

**DOI:** 10.1186/1748-7161-10-S1-O20

**Published:** 2015-01-19

**Authors:** Ka Yee Cheuk, Tsz Ping Lam, Lyn Lee Ning Wong, Alec Lik Hang Hung, Arthur Fuk Tat Mak, Kwong Man Lee, Bobby Kin Wah Ng, Jack Chun Yiu Cheng

**Affiliations:** 1 Department of Orthopaedics and Traumatology, The Chinese University of Hong Kong, Hong Kong; 2 Joint Scoliosis Research Center of the Chinese University of Hong Kong and Nanjing University, China; 3 Department of Mechanical and Automation Engineering, The Chinese University of Hong Kong, Hong Kong; 4 Lee Hysan Clinical Research Laboratories, The Chinese University of Hong Kong, Shatin, Hong Kong

## Objectives

Although AIS was associated with low bone mass, reports on bone mechanical properties in AIS are sparse. The objective of this study is to evaluate bone mechanical properties with finite element analysis (FEA) using in-vivo High-Resolution Peripheral Quantitative Computed Tomography (HR-pQCT) in AIS and compare that with normal controls.

## Material and methods

97 AIS girls and 99 female controls between 11-14 years old were recruited. Dietary calcium intake and physical activity level were assessed with a standard Food Frequency Questionnaire and the Modified Baecke Questionnaire respectively. With HR-pQCT, an established model on morphology and micro-structure of the non-dominant distal radius was generated for FEA in terms of Stiffness, Failure Load and Apparent Modulus. Multivariate linear regression analysis was used to investigate the difference between AIS and controls after adjusting for age in Model 1 and for age, calcium intake and physical activity level in Model 2.

## Results

2-tailed Student's t-test showed AIS subjects had lower Stiffness, lower Failure Load and lower Apparent Modulus when compared with normal controls (% difference = -6.81%, -7.10% & -8.10% respectively, all with p<0.05). AIS girls had lower Failure Load (B=-136.0, p=0.04) and lower Apparent Modulus (B=-146.2, p=0.021) in Model 1 with adjustment for age. In Model 2, difference in Apparent Modulus remained statistically significant with AIS being associated with lower Apparent Modulus after adjustment for age, calcium intake and physical activity level (B=-137.1, p=0.037).

## Conclusions

Higher Stiffness, higher Failure Load and higher Apparent Modulus mean better resistance to deforming forces. Crude comparison indicated AIS was associated with lower Stiffness, lower Failure Load and lower Apparent Modulus. Analysis with Model 1 showed that the difference in Stiffness was due to confounding from age. Further analysis with Model 2 indicated the difference in Failure Load could arise from difference in calcium intake and physical activity level between AIS and controls. Notably AIS remained associated with lower Apparent Modulus after adjusting for age, calcium intake and physical activity level. This indicated the presence of an underlying biochemical or biomechanical mechanism yet to be identified that could be responsible. Further studies on this area are warranted in order to gain in-depth understanding of the nature of low bone mass and bone strength and their roles in the etiopathogenesis of AIS.

This study was supported by Research Grants Council of the Hong Kong S.A.R., China (Project no: 468809 and 468411).

